# Kinase inhibitors allosterically disrupt a regulatory interaction to enhance PKCα membrane translocation

**DOI:** 10.1016/j.jbc.2021.100339

**Published:** 2021-01-26

**Authors:** Lisa G. Lippert, Ning Ma, Michael Ritt, Abhinandan Jain, Nagarajan Vaidehi, Sivaraj Sivaramakrishnan

**Affiliations:** 1 Department of Genetics, Cell Biology, and Development, University of Minnesota, Minneapolis, Minnesota, USA; 2 Department of Computational and Quantitative Medicine, Beckman Research Institute of the City of Hope, Duarte, California, USA; 3 The Jet Propulsion Laboratory, California Institute of Technology, Pasadena, California, USA

**Keywords:** allosteric regulation, kinases, inhibitor, protein translocation, PKCα, PKC, DAG, diacylglycerol, DMSO, dimethyl sulfoxide, FBS, fetal bovine serum, G-loop, Gly-rich loop, LPA, lysophosphatidic acid, PKCα, protein kinase Cα, PS, pseudosubstrate, REU, Rosetta energy units, SMKI, small molecule kinase inhibitor

## Abstract

The eukaryotic kinase domain has multiple intrinsically disordered regions whose conformation dictates kinase activity. Small molecule kinase inhibitors (SMKIs) rely on disrupting the active conformations of these disordered regions to inactivate the kinase. While SMKIs are selected for their ability to cause this disruption, the allosteric effects of conformational changes in disordered regions is limited by a lack of dynamic information provided by traditional structural techniques. In this study, we integrated multiscale molecular dynamics simulations with FRET sensors to characterize a novel allosteric mechanism that is selectively triggered by SMKI binding to the protein kinase Cα domain. The indole maleimide inhibitors BimI and sotrastaurin were found to displace the Gly-rich loop (G-loop) that normally shields the ATP-binding site. Displacement of the G-loop interferes with a newly identified, structurally conserved binding pocket for the C1a domain on the N lobe of the kinase domain. This binding pocket, in conjunction with the N-terminal regulatory sequence, masks a diacylglycerol (DAG) binding site on the C1a domain. SMKI-mediated displacement of the G-loop released C1a and exposed the DAG binding site, enhancing protein kinase Cα translocation both to synthetic lipid bilayers and to live cell membranes in the presence of DAG. Inhibitor chemotype determined the extent of the observed allosteric effects on the kinase domain and correlated with the extent of membrane recruitment. Our findings demonstrate the allosteric effects of SMKIs beyond the confines of kinase catalytic conformation and provide an integrated computational-experimental paradigm to investigate parallel mechanisms in other kinases.

Protein kinases catalyze the transfer of a phosphate group from ATP to a protein substrate (1). Kinase activity relies on the spatial coordination of nonconsecutive residues in the kinase domain, which form conserved structural motifs termed spines surrounding the ATP-binding sites (2). Pharmacological modulation of kinase activity is almost exclusively achieved through small molecule kinase inhibitors (SMKIs) that target the conserved kinase domain (3). SMKIs are often selected for their ability to disrupt spines and consequently abolish kinase activity. However, recent studies suggest that the entire eukaryotic kinase domain functions as an extended allosteric network, with groups of amino acid residues distal to the nucleotide-binding site working in concert to determine kinase activity (2). Hence, in addition to impacting spines, it is likely that SMKIs can influence kinase-substrate and kinase-regulatory interactions through allosteric coupling between the nucleotide site and distal protein interaction interfaces in the kinase domain. However, the allosteric effects of SMKIs on intramolecular and intermolecular interactions in protein kinases remain broadly uncharacterized. In this study, we examine the allosteric effects of SMKIs on a model multidomain kinase, protein kinase Cα (PKCα).

PKCα is a member of the AGC kinase superfamily (4) and is involved in a number of processes including cell growth, transcriptional regulation, and immune response (5). Given its nodal regulation of numerous signaling cascades (6, 7), PKC has been a prominent target of small molecule therapeutics (8, 9, 10). Efforts to selectivity target PKC isoforms have yielded a range of ATP-competitive SMKIs, some of which have advanced through clinical trials. Nonetheless, selective targeting of the pathological functions of PKCs is complicated by the diversity of protein substrates they phosphorylate and their multiplexed regulation through intramolecular and intermolecular interactions in response to a range of upstream stimuli (11). Further, there is currently no complete structure of any PKC isoform, and there is limited high-resolution information on the PKC-substrate interaction (12, 13). To address this knowledge gap, we focus our study on an intramolecular regulatory interaction in PKCα involving the C1a and kinase domains that we previously demonstrated to be essential for basal autoinhibition of kinase activity (14). Here, we used multiscale MD simulations, extensively validated by experimental FRET sensor measurements, to characterize the C1a-kinase domain binding interface.

PKC stimulation with a combination of Ca^2+^, diacylglycerol (DAG), and phorbol ester (*e.g.*, PMA) results in membrane translocation and kinase activation. Activated PKC is known to self-associate through intramolecular and intermolecular interactions between the regulatory and catalytic domains (15, 16). PKC oligomers display nonuniform dispersity that can be influenced by the combination of activating stimuli (Ca^2+^, PIP_2_, DAG, PMA). Hence, structural characterization of PKC oligomers has been challenging. Nonetheless, multiple reports have documented their formation in live cells (16, 17), and disrupting self-association with cell penetrant peptides has been shown to selectively block PKC-stimulated ERK1/2 phosphorylation (15). In addition, we have previously shown that the SMKI BimI enhances PKCα self-association with consequent prolonged membrane localization following lysophosphatidic acid (LPA) stimulation (15). However, the relative allosteric effects of SMKIs on PKC oligomerization remain unexplored.

In this study, we characterize a novel allosteric mechanism that is selectively triggered by SMKI binding to the PKCα kinase domain. The indole maleimide inhibitors BimI and sotrastaurin displace the Gly-rich loop (G-loop) that normally shields the ATP-binding site. Displacement of the G-loop interferes with a structurally conserved binding pocket at the N-terminus of the kinase domain. This binding pocket in PKCα is occupied by the C1a regulatory domain, where it masks a DAG binding site. BimI/sotrastaurin binding displaces the G-loop, releasing C1a and exposing the DAG binding site. Engaging this allosteric mechanism leads to enhanced PKCα translocation both to synthetic lipid bilayers and to live cell membranes in the presence of DAG. Inhibitor chemotype determines the extent of allosteric effects on the kinase domain, in turn correlating with the pattern of membrane recruitment. Our findings demonstrate the allosteric effects of SMKIs, beyond the confines of kinase catalytic conformation and provide an integrated computational-experimental paradigm to investigate parallel mechanisms in other kinases.

## Results

### Select ATP-competitive SMKIs displace regulatory-kinase domain interactions

**Figure 1:**
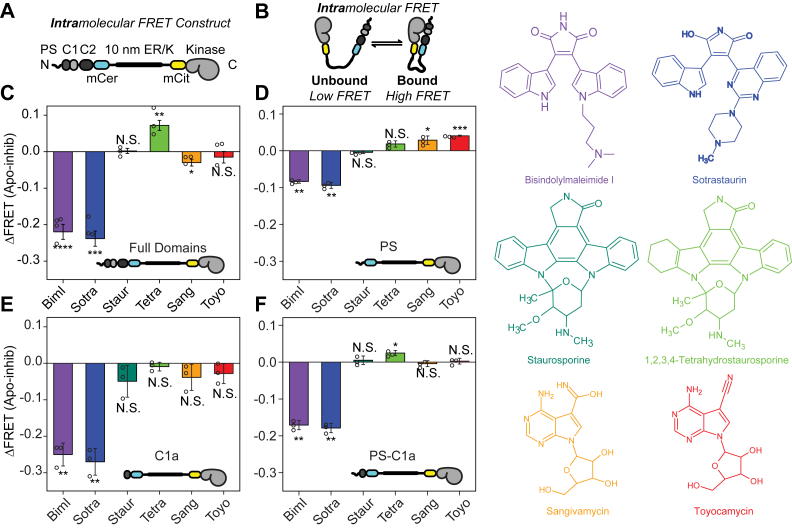
**Inhibitors alter PKCα regulatory interactions *in vitro.****A*, schematic of constructs used to measure intramolecular interactions. A 10 nm ER/K linker flanked by an mCerulean (mCer, donor) mCitrine (mCit, acceptor) FRET pair was inserted into PKCα in between the N-terminal regulatory domains and the C-terminal kinase domain. *B*, interaction affinity between domains was measured by the FRET ratio as depicted. *C*–*F*, ΔFRET levels of the depicted constructs in the presence of the indicated inhibitors. ΔFRET is the change in FRET ratio relative to the no inhibitor condition (apo). BimI: Bisindolylmaleimide I, Sotra: sotrastaurin, Staur: staurosporine, Tetra: 1,2,3,4-tetrahydrostaurosporine, Sang: sangivamycin, Toyo: toyocamycin. Error bars show standard deviation. N ≥ 3 measurements from different protein preparations. Individual data points are represented by circles on their respective bars. Significance was determined using a paired *t* test of the raw FRET ratios relative to no inhibitor where ∗∗∗∗ indicates *p* < 0.0001, ∗∗∗ indicates *p* < 0.001, ∗∗ indicates *p* < 0.01, ∗ indicates *p* < 0.05, and N.S. denotes *p* ≥ 0.05. PKCα, protein kinase Cα.

We have previously shown that ATP-competitive SMKIs can allosterically influence the kinase-substrate interaction in PKCα (18). Here, we used previously reported SPASM PKCα sensors (15) (Fig. 1*A*) to examine the influence of SMKIs on interactions between the kinase and regulatory domains of PKCα. The sensors contain a single α-helical ER/K linker flanked by a FRET donor/acceptor pair that sets the effective concentration of the intramolecular interaction (19). FRET ratio in these constructs (Fig. 1*B*) correlates linearly with the fraction of molecules in the bound state (19). We found that both the ATP-analog inhibitors (sangivamycin and toyocamycin) and staurosporine derivatives (staurosporine and its analog tetrahydrostaurosporine) have minimal effects on the regulatory-kinase domain interaction as reported by changes in the FRET ratio (ΔFRET; Fig. 1*C*). In contrast, the indole maleimide inhibitors, BimI and sotrastaurin, substantially inhibited this interaction. Interestingly, the effects of this panel of inhibitors on the kinase-regulatory domain interaction recapitulate the results from our previous report on the kinase-substrate peptide interaction (18). Owing to this correlation, we tested the effects of the inhibitor on the interaction between the kinase and a pseudosubstrate peptide (PS peptide) at the N-terminus of the regulatory domain. The PS peptide binding showed a similar inhibition profile as the complete regulatory domain, albeit with smaller changes in FRET ratio (Fig. 1*D*).

We have previously reported that the C1a regulatory domain has a strong interaction with the kinase domain that is essential for basal autoinhibition of PKCα kinase activity (14). Hence, we next examined the effects of inhibitors on the C1a-kinase domain interaction. BimI and sotrastaurin showed robust inhibition of the C1a-kinase domain interaction (Fig. 1*E*), and the two domains (PS and C1a) together showed similar changes in FRET ratio as the complete regulatory domain (Fig. 1*F*). While we have previously established that the allosteric effects of BimI on substrate binding is mediated by changes in conformation of the kinase domain activation loop (18), the structural basis for inhibition of C1a binding remained unclear.

### *De novo* identification and validation of the C1a-kinase domain–binding interface

**Figure 2:**
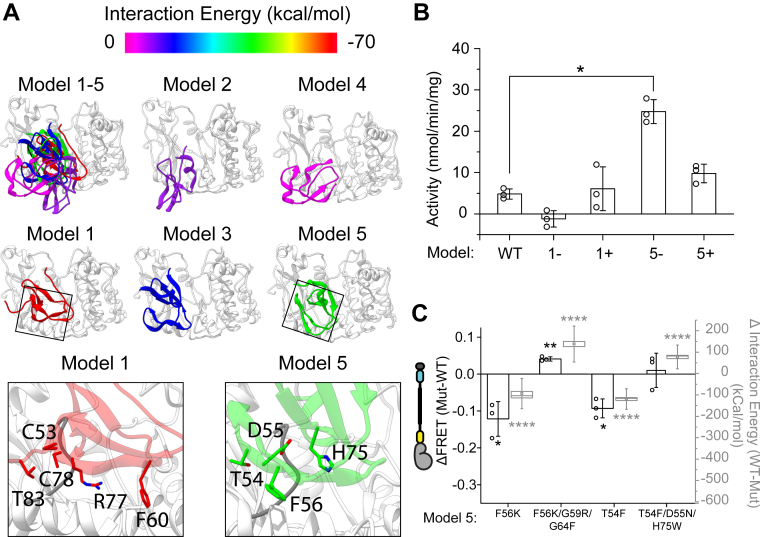
**Modeling C1a-Kinase interactions.***A*, five structural models of C1a domain interactions with the kinase domain obtained from the coarse resolution torsional GNEIMO-REMD simulations. Interactions are color-coded according to interaction energy. A close up view of the residues in C1a interacting with the G-loop in the two models (models 1 and 5) with the most favorable interaction energy (*bottom, black boxes*). C1a interacts with G-loop residues from N-lobe of the kinase domain. *B*, activity measurements of full length PKCα with mutations derived from the indicated models (+: stabilizer; -: destabilizer). Model 1-: C53E/G61W/C78D, 1+: F60D/R77D/T83K, 5-: F56K/G59R/G64F, 5+: T54F/D55N/H75W. *C*, FRET sensor measurements (*black outlined bars*, *left axis*) on single and combination mutations derived from model 5 on four C1a residues that are predicted to interact with the kinase domain. The calculated difference in average interface interactions energies from the all-atom MD simulations between wildtype (WT) and the mutant using model 5 are shown in *light gray box* and *whisker plots* (*right axis*). These difference in interaction energies correlate well with the measured FRET intensity changes upon mutation. For activity and FRET measurements, N ≥ 3 measurements from different protein preparations. Individual data points are represented by *circles* on their respective bars. For modeling data in figure *B*, the interaction energies were calculated from mutations performed using Rosetta (see Experimental procedures). For the interface interaction energies shown in figure *C*, we used the mmpbsa method that includes desolvation penalty, with the 52 evenly spaced snapshots extracted from all-atom MD simulations (see Experimental procedures). Bar graphs are average and standard deviation, *box* and *whisker data* are depicted as follows: *midline* is median, *spot* is mean, *box* is SEM, and *whiskers* are standard deviation. For activity data, significance was determined *via* a two-way ANOVA, comparing the effects of lipids and the various sets of mutations followed by Tukey’s post-hoc significance test. For FRET and interaction energy, significance was calculated by, respectively, either a paired or unpaired Student’s *t* test against no WT protein. Significance is indicated on the plot where ∗∗∗∗ indicates *p* < 0.0001, ∗∗ indicates *p* < 0.01, ∗ indicates *p* < 0.05, and N.S. denotes *p* ≥ 0.05. PKCα, protein kinase Cα.

The single, partial structure of a classical PKC (PKCβII) does not resolve the potential binding interface for C1a on the kinase domain (20). Hence, we first generated a *de novo* model of the C1a-kinase domain interaction. Briefly, the known interaction interfaces for PS and C1b were used as constraints in GNEIMO-MD simulations to identify potential interaction sites for C1a. The C1a domain was modeled as linked to the known interfaces of pseudo substrate and C1b, with native flexible linkers V1 and V1’, and positioned 35 Å from the kinase domain (Fig. S1*A*). GNEIMO-MD simulations were used to predict possible binding sites for the C1a domain on the kinase domain under the constraints from two flexible linkers. The snapshots of MD simulation trajectories representing the potential interacting sites of C1a were grouped into conformational clusters, and the top four conformational clusters were rank ordered by Rosetta energy units (REU; Fig. S1*B*). The most energetically favored cluster was further analyzed to identify the top five scoring structures based on energy of the C1a-kinase domain complex (Fig. S1*C*; Fig. 2*A*). Model 1 and 5 were selected for experimental testing based on interface interaction energy (Fig. S1*D*) and Rosetta mutagenesis (Fig. S1*E*). Three single point mutations that showed gain in interface energy upon mutation and three single point mutations that led to weakening of the interface energy all calculated using Rosetta energy function upon mutation in model 1 and model 5 were independently identified (Fig. 2*A* insets; Fig. S1*F*). For model 1, C53E/G61W/C78D were identified as stabilizing (9 REU lower than WT) whereas F60D/R77D/T83K were identified as destabilizing (22 REU higher than WT). For model 5, T54F/D55N/H75W were identified as stabilizing (10 REU lower than WT), and G64F/G59R/F56K were identified as destabilizing (10 REU higher than WT). Stabilizing and destabilizing mutations for model 1 and 5 were assessed in an *in vitro* kinase activity assay (Fig. 2*B*). Destabilizing mutations in model 5 resulted in a significant increase in *in vitro* kinase activity, consistent with the release of basal autoinhibition in PKCα, suggesting model 5 as the putative interaction site. The Rosetta-based interface energy calculation (REU) is a crude measure that does not take into account the explicit desolvation energies. Therefore, the predictions of the effect of both destabilizing and stabilizing mutations affected the kinase activity but did not correlate with the activity as predicted. However, the average C1a–CD interface energy calculated for the mutants using the snapshots from all-atom MD simulations performed in explicit solvent agreed well with the FRET measurements as shown in Figure 2*C*.

To test the effect of these mutations on the strength of the C1a interaction with the kinase domain (and not kinase activity), we used FRET sensor measurements (Fig. 2*C*, *left axis*). To refine the structural models of C1a kinase domain interactions extracted from GNEIMO torsion MD simulations, we performed all-atom MD simulations on model 5 and the mutants shown in Figure 2*C*. Using the snapshots from the all-atom MD simulations for the mutants and the WT, we calculated the average difference in the C1a-kinase domain interaction energies between the WT and the mutant as shown in Figure 2*C* (*right axis*). These more accurate interface interaction energies calculated using all-atom force fields in explicit solvent correlate well with the changes in FRET intensities, as measured using C1a-kinase domain FRET sensors (Fig. 2*C*).

### Identification of a PKC-conserved druggable binding pocket on the kinase domain

**Figure 3:**
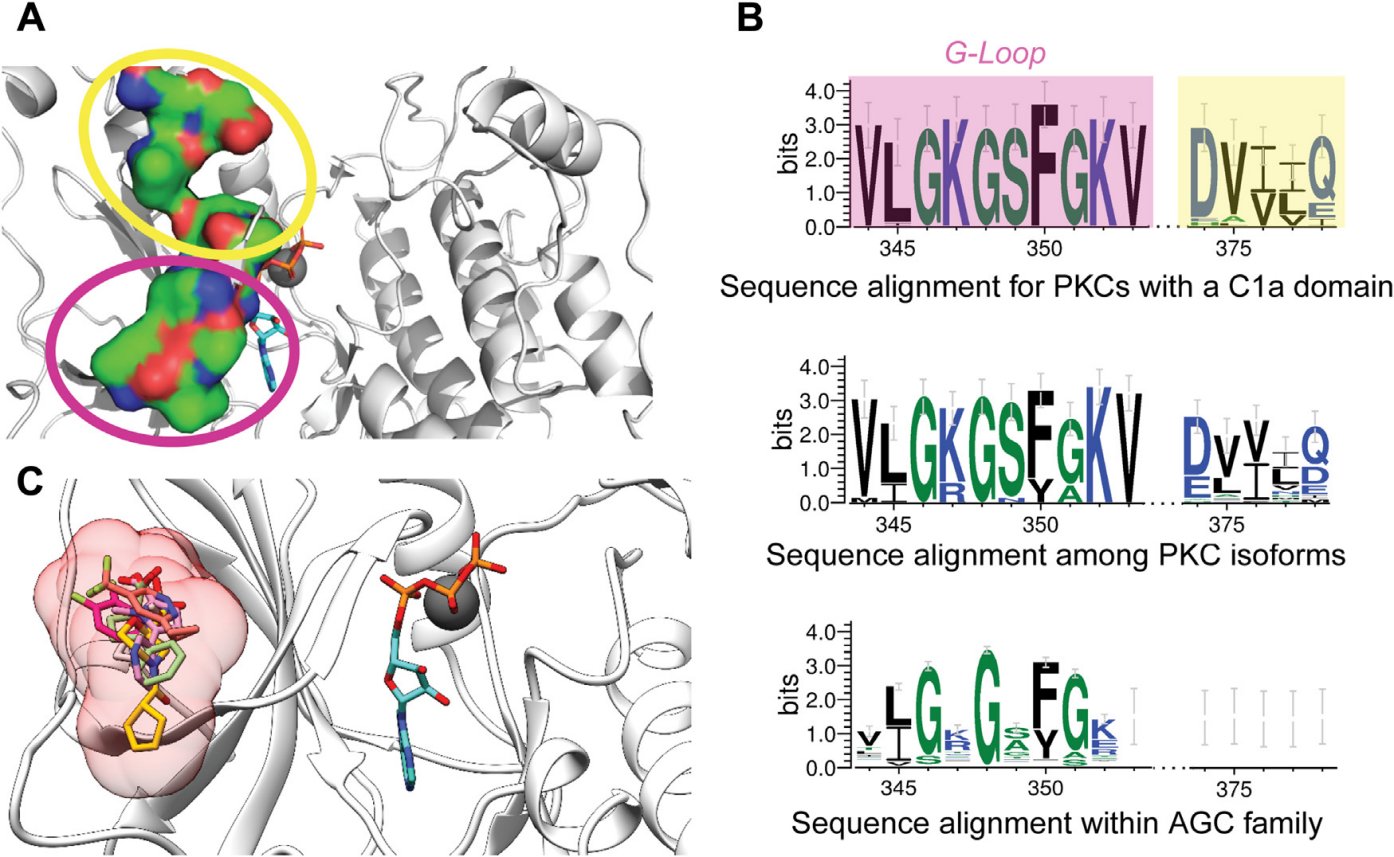
**Sequence conservation and consensus of residues on the kinase domain that interface with C1a.***A*, surface representation of kinase domain residues that interact (<4.5 Å between nonhydrogen atoms colored as **CON**) with C1a. Residues circled in *purple* are the G-loop. Only the residues in the kinase domain and not the C1a domain are shown here. *B*, WebLogo representation of sequence conservation of residues highlighted in (*A*). Residues conserved in the PKC subfamily are also responsible for C1a binding. Shaded in residues in the *top panel* correspond to their respectively *colored circles* in (*A*). *C*, six representative small-molecule fragments from the Zinc database identified using *Find Binding Site* in stick representation with surface representation of the ensemble (*pink*). PKC, protein kinase C.

Residues on the kinase domain that interface with C1a form a binding pocket encompassing the G-loop (G-loop pocket; Fig. 3*A*). Sequence conservation analysis of the interface residues suggests that they are highly conserved across all PKCs with a C1a domain, further validating them their role in C1a engagement (Fig. 3*B*, *top panel*). All classical and novel PKC isoforms contain a C1a domain (11). Accordingly, kinase domain residues are conserved across the PKC superfamily (Fig. 3*B*, *middle panel*). In contrast, with exception to the eukaryotic kinase-conserved GxGxxG motif, the kinase-C1a interface residues are not conserved across AGC kinases, of which PKCs are a subfamily (Fig. 3*B*, *bottom panel*). These data suggest that the C1a binding pocket can be used to selectively target PKC function. Independently, the *FindBindSite* (FBS) technique (see Experimental procedures (21)) identified the G-loop binding pocket among the top five binding interfaces, including the ATP-binding site (Fig. S2). The G-loop pocket is capable of accommodating a wide array of small molecules (Fig. S2; Fig. 3*C*), suggesting that it is a promising location to allosterically modulate PKCα kinase function.

### Select ATP-competitive SMKIs displace the G-loop to allosterically modulate the C1a-kinase domain interaction

**Figure 4:**
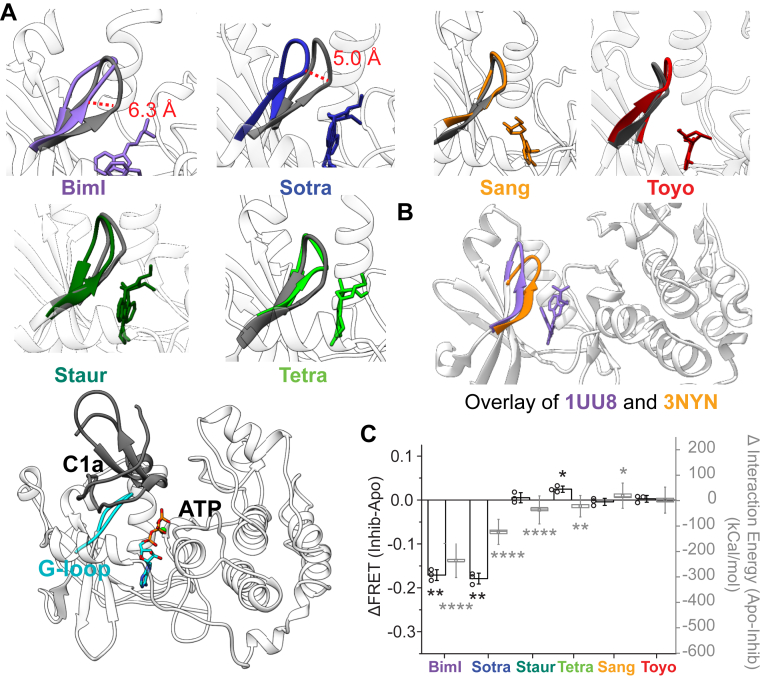
**Allosteric effects of SMKIs on G-loop position.***A*, six structural models of G-loop position extracted from all-atom MD simulations of inhibitor-bound PKCα (*colored loops*) as compared with the nucleotide-free (Apo) kinase domain (*gray loops*). The six structures are representative conformations extracted from the most occupied conformation cluster (see Experimental procedures). The *bottom panel* depicts the orientation of the proposed C1a domain interaction in relation to the G-loop when bound to ATP. *B*, overlay of two previously published crystal structures of kinases highly related to PKC bound to BimI (PDK1, 1UU8, *purple*) or sangivamycin (GRK6, 3NYN, *orange*) depicting the shift in the position of the G-loop. *C*, the extent of weakening of C1a binding to the kinase domain by several SMKIs tested by FRET sensors (*black outline bars*, *left axis*). The calculated C1a-kinase domain interaction energies in the presence of SMKIs (*light grey box* and *whisker*, *right axis*) show good correlation with the FRET measurements. For activity and FRET measurements, N ≥ 3 measurements from different protein preparations were used. Individual data points are represented by *circles* on their respective bars. The interaction energies were calculated from all-atom MD simulation trajectories for Apo and indicated inhibitors using 52 evenly spaced snapshots extracted from MD simulations and using mmpbsa energy module (N = 52 snap shots evenly spaced from 0.5 to 1 μs). Box and whisker plots are depicted as follows: midline represents median, symbol represents mean, box represents SEM, and whiskers represent SD. For FRET and interaction energy, significance was calculated by, respectively, either a paired or unpaired Student’s *t* test against no wildtype protein. Significance is indicated on the plot where ∗∗∗∗ indicates *p* < 0.0001, ∗∗ indicates *p* < 0.01, ∗ indicates *p* < 0.05. and no symbol indicates no significance. PKCα, protein kinase Cα.

The GxGxxG motif within the G-loop is a highly conserved sequence motif in eukaryotic kinases. In our MD simulations, the G-loop was found to shield ATP from solvent suggesting an allosteric link between the C1a and ATP-binding sites. To address this possibility, the experimentally validated model of the C1a-kinase domain interaction was used in MD simulations that examine the impact of the six different ATP-site kinase inhibitors. Simulations revealed that the BimI analogs (Sotra and BimI) displace the G-loop away from the nucleotide binding pocket, which in turn interferes with the kinase-C1a binding interface (Fig. 4*A*). In contrast, neither the staurosporine analogs (Staur and Tetra) nor the ATP analogs (Sang and Toyo) have significant effects on G-loop conformation relative to the nucleotide-free (Apo) state (Fig. 4*A*). Interestingly, the distinct conformational states of the G-loop observed in our MD simulations of the PKCα kinase domain in the presence of BimI and sangivamycin are also apparent in published crystal structures of PDK1 complexed with BimI (1UU8) and GRK6 complexed with Sang (3NYN) (Fig. 4*B*). These comparisons suggest that the inhibitor-triggered G-loop conformational states are prevalent across AGC kinases. To quantify the effects of inhibitors on C1a binding, change in interface binding energies relative to the nucleotide-free (Apo) state were computed (Fig. 4*C*). Changes in interface binding energy were consistent with changes in the strength of interaction measured as reported by FRET ratio for all six inhibitors tested in this study (Fig. 4*C*).

### Simulations suggest that inhibitor displacement of the C1a domain unmasks a diacylglycerol binding site

A key step in PKC activation is its recruitment to cell membranes through C1a/b interactions with DAG (11). The C1a domain contains a DAG binding site, which facilitates PKC membrane recruitment following activation. In the nucleotide-free (Apo) state of the kinase domain, MD simulations reveal that residues essential for DAG binding (F56 and W58) (11) are buried in the C1a-kinase domain interface (Fig. S3*A*) and further shielded from solvent by the PKCα N-terminus (Fig. S3, *B* and *C*). These simulations further suggest that BimI binding to the kinase domain displaces C1a, which in turn alters the conformation of the V1 variable linker and the PKCα N-terminus (Fig. S3*B*). The combined effect of these conformational changes is a model that hints at solvent exposure of the C1a DAG binding site (Fig. S3*C*). The exposure of this DAG binding site would suggest that inhibitor treatment would enhance membrane-kinase interactions, which are a hallmark of PKC activation.

### SMKIs differentially influence PKCα homo-oligomerization

We have previously shown that PKCα activation results in its homo-oligomerization with nanomolar affinity, both *in vitro* and in live cell membranes (14, 15). Our previous study also shows that BimI prolongs PKCα localization to live cell membranes following LPA stimulation (15). We therefore wanted to explore whether the SMKI-induced membrane localization we have observed may also be influenced by oligomerization of the kinase. Hence, we used a bi-molecular FRET sensor combination (mCer-PKCα and mCit-PKCα) to examine the effects of SMKIs on PKCα homo-oligomerization (Fig. S4*A*). Under activating conditions (3.2 μM PMA and 1.5 mM CaCl_2_), both BimI-like inhibitors (BimI and sotrastaurin) and staurosporine-like inhibitors (staurosporine and tetrahydrostaurosporine) showed substantial increases in homo-oligomerization of recombinant PKCα relative to the Apo state (Fig. S4*B*). In contrast, ATP-analog inhibitors (sangivamycin and toyocamycin) caused a modest decrease in homo-oligomerization (Fig. S4*B*).

### SMKIs influence PKCα membrane recruitment to synthetic lipid bilayers and live cells

**Figure 5:**
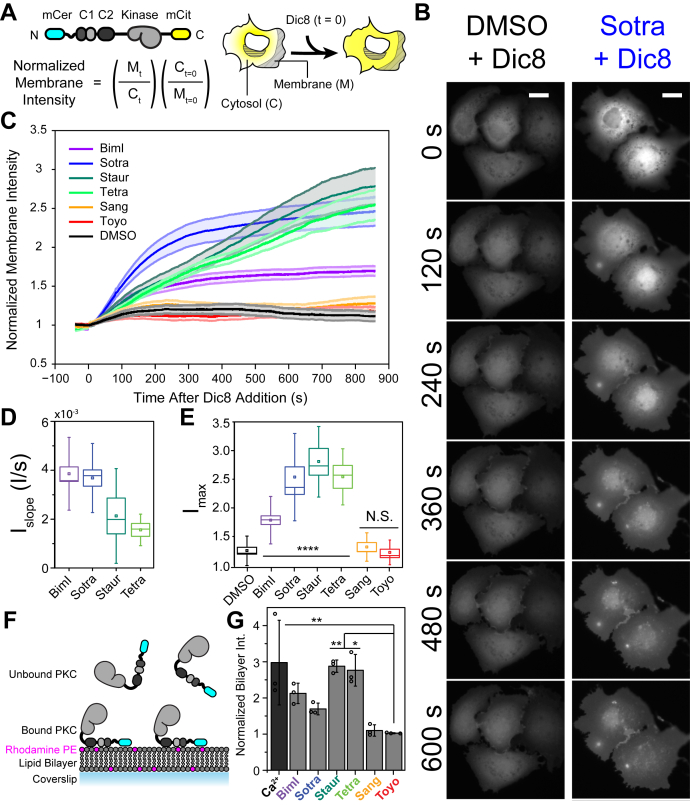
**Inhibitors impact spatial-temporal localization of PKCα.***A*, schematic of live cell translocation assay and analysis method. Fluorescent PKCα (*yellow*) translocations from the cytoplasm to the membrane upon stimulation with DiC_8_. Membrane localization is quantified by the ratio of the membrane (M) and cytoplasmic (C) intensities at time t (s) and normalized to the ratio immediately prior to stimulation (t = 0). *B*, frames from representative movies of PKCα translocation with either DMSO or Sotra. Translocation is marked by a decrease in fluorescence in the perinuclear cytoplasmic and a corresponding increase at the cell periphery. *C*, membrane localization of PKCα in COS-7 cells in the presence of six ATP-competitive PKC inhibitors or DMSO following stimulation with DiC_8_. *Shaded regions* denote standard error of the mean (SEM) N ≥ 4 dishes with at least 6 cells per condition. *D*, initial rate of membrane translocation (I_slope_) in the presence of ATP-competitive inhibitors. I_slope_ is quantified as the slope of a linear fit to the normalized intensity curve over the first 100 s following stimulation. Data are plotted only for robustly translocating inhibitors because rates for modestly translocating conditions (DMSO, Sang, Toyo) were unreliable and heavily influenced by small fluctuations. Negative values were not included in the analysis. *E*, maximum membrane intensity under the indicated conditions. *D*, schematic of *in vitro* membrane recruitment reconstitution assay. Supported lipid bilayers containing 5% diacylglycerol, 69.9% 1,2-dioleoyl-sn-glycero-3-phosphoethanolamine, 25% 1,2-dioleoyl-sn-glycero-3-phospho-L-serine, and 0.1% fluorescent PE (*magenta*) were formed on a glass coverslip. Membrane recruitment was measured as the intensity of fluorescent PKCα at the bilayer surface. *E*, *in vitro* membrane recruitment in the presence of inhibitors or calcium (positive control). Intensities are normalized to an ATP-only condition. N ≥ 3 membranes using different lipid and protein preparations. Individual repeats are represented as *circles* on their respective bars, and errors are presented as standard deviation. Box and whisker plots are used where displaying the individual repeats is impractical. For box and whisker plots, midline represents median, spot represents mean, box represents SEM, and whiskers represent SD. Statistical comparisons were made using a paired Student’s *t* test. Significance is indicated on the plot where ∗∗∗∗ indicates *p* < 0.0001, ∗∗ indicates *p* < 0.01, ∗ indicates *p* < 0.05, and N.S. or no symbol indicates no significance. PKCα, protein kinase Cα.

To address the impact of inhibitors on membrane recruitment, mCitrine-PKCα was transiently expressed in COS7 mammalian cells, and its membrane translocation was measured in response to stimulation with 1,2-dioctanoyl-*sn*-glycerol (DiC_8_), a DAG analog (Fig. 5*A*). Following stimulation, PKCα translocated from the cytoplasm to the cell membrane, as seen by an increase in the membrane intensity (Fig. 5*B*). The six different inhibitors showed marked differences in the shapes of their normalized membrane intensity profiles (Fig. 5*C*). While addition of BimI and sotrastaurin resulted in a rapid initial translocation followed by a plateau, staurosporine and tetrahydrostaurosporine resulted in a slower rate of translocation. These differences were quantified using the maximum membrane intensity (I_max_) and the initial slope of the intensity curve (I_slope_) (Fig. 5, *D* and *E*). I_max_ is defined as the highest point on the membrane intensity curve and is a measure of the total amount of PKCα recruited to the membrane. For traces that did not plateau within the 15-min imaging period, this is equal to the intensity of the final frame. Correspondingly, I_slope_ is the initial rate of translocation, determined from a linear fit to the intensity for 100 s following stimulation and normalized to the maximum intensity to account for differences in I_max_. A larger I_slope_ indicates faster translocation. Because two of the inhibitors, sangivamycin and toyocamycin, and the dimethyl sulfoxide (DMSO) control resulted in very weak translocation, I_slope_ values for these conditions are unreliable and are therefore not shown. The remaining four inhibitors significantly enhanced maximal membrane recruitment (I_max_) with the aggregate rates of BimI-like inhibitors (BimI and sotrastaurin) greater than staurosporine-like inhibitors (staurosporine and tetrahydrostaurosporine). Taken together, these data demonstrate the differential effects of SMKIs on membrane localization of activation PKCα.

Given that membrane recruitment of PKCs in cells could be influenced by a number of factors, including membrane-localized proteins, we examined the intrinsic effects of SMKIs using synthetic lipid bilayers. Supported lipid bilayers mimicking the composition of cellular membranes were prepared on coverslips (Fig. 5*F*). Additionally, these lipid bilayers contain rhodamine-PE to aid in the evaluation of membrane integrity (see Experimental procedures). mCer-PKCα was recruited to lipid bilayers to varying extents in the presence of ATP or six SMKIs (Fig. 5*G*). Lipid recruitment was quantified from fluorescence intensity normalized to the ATP condition. Recruitment with 2 mM CaCl_2_, a known driver of PKCα lipid binding, was used as a positive control. Consistent with live cell observations, the nucleotide analogs toyocamycin and sangivamycin were indistinguishable from the ATP control. In contrast, BimI-like and staurosporine-like inhibitors significantly enhanced surface recruitment, with staurosporine-like inhibitors performing the best overall. These *in vitro* studies support the intrinsic effects of SMKIs on membrane recruitment of PKCα.

## Discussion

**Figure 6:**
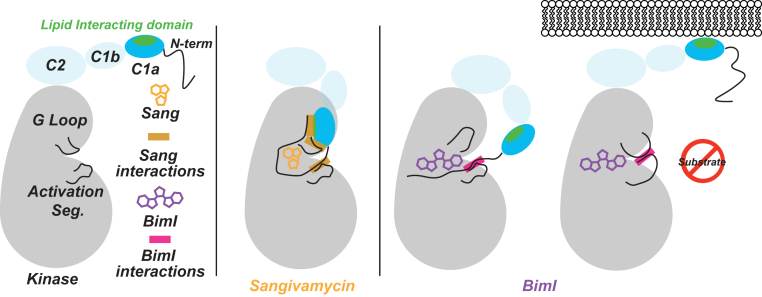
**Allosteric effects of small-molecule kinase inhibitors in PKCα.** ATP-analog sangivamycin retains an autoinhibitory interaction between the kinase domain, C1a domain, and N-terminal fragment. In contrast, BimI displaces the G-loop, which interferes with C1a binding to a conserved binding pocket in the N-lobe of the kinase domain. Release of the C1a domain exposes the diacylglycerol binding interface (*green*), resulting in PKCα translocation to cell membranes in live cells. An intramolecular interaction between the G-loop and activation segments blocks substrate binding to the kinase domain. PKCα, protein kinase Cα.

The eukaryotic kinase domain contains a constellation of nonconsecutive amino acid residues, termed spines, whose relative spatial arrangement is critical for the catalytic transfer of phosphate from ATP to a protein substrate. Several of these residues reside in two unstructured segments, the G-loop and the activation segment. To achieve the structural alignment of spines necessary for catalysis, the G-loop and the activation segment adopt similar “active” conformations in catalytically active kinases. The mechanism of inhibition by SMKIs often relies on disrupting this structural alignment of spines to trap the kinase in an inactive conformation. While SMKIs are often selected for their ability to cause this disruption, interpreting inhibitor-specific conformations is limited by the lack of dynamic information provided by traditional structural techniques. Here, we leverage multiscale MD and FRET sensors to highlight the allosteric effects of SMKIs driven by conformational effects on unstructured loop segments. Specifically, the G-loop was found to hover over the nucleotide binding site, shielding ATP from solvent. Our MD simulations in PKCα reveal that SMKIs such as BimI hit the “roof” of the ATP binding site and therefore displace the G-loop. In contrast, we found that ATP analog inhibitors and staurosporine-like inhibitors draw the G-loop into the catalytic site akin to ATP. The behavior of the G-loop observed in the PKCα MD simulations is consistent with two previously reported X-ray crystal structures of AGC kinases (22, 23). The G-loop of PDK1 was found to adopt a similar conformation relative to the catalytic site when bound to BimI (22). Similarly, the G-loop of GRK6 was drawn into the catalytic site when bound to the ATP analog sangivamycin (23). In our simulations with PKCα, we found that the displaced G-loop interferes with a conserved small-molecule binding pocket in the kinase domain. In PKCα, the C1a-kinase domain interaction interface overlaps with this binding pocket. During the dynamics simulations, we observed that BimI and sotrastaurin displaced the G-loop, which in turn weakened the C1a interaction with the kinase domain. In contrast, staurosporine and tetrahydrostaurosporine had much weaker interactions with the G-loop, and consequently, dynamics simulations did not show displacement of either the G-loop or C1a domain. Our dynamics simulations also suggest ripple effects of C1a displacement on the V1 linker, PS, and N-terminal region of PKCα. The net conformational effect of this rearrangement is the unmasking of two key residues, F56 and W58, in the C1a domain that comprise the DAG binding site (24). These coordinated changes in the regulatory-kinase domain interaction parallel dramatic changes in the spatial-temporal pattern of activated PKCα localization in live cells. Taken together, our findings expand on the binary active/inactive view of structural conformations within the kinase domain to include mixed conformations triggered by SMKIs (Fig. 6).

Multidomain kinases such as PKCα often contain a series of regulatory domains that are tethered to a conserved kinase domain by unstructured linkers. The dynamic, multiplexed nature of kinase-regulatory interactions has limited high-resolution structural information on kinase-regulatory domain interfaces. There are as yet no full-length structures of any PKC isoform. In the absence of such structural information, this study engaged multiscale MD simulations validated by FRET sensor measurements to identify the structural interface of a key regulatory-kinase domain interaction in PKCα. The multiscale MD simulations leveraged structural information on an anomalous high affinity kinase-substrate peptide interaction in PKCiota to reconstruct the regulatory interaction between PKCα and its PS peptide. In full-length PKCα, the PS peptide is followed by an unstructured linker and subsequently the C1a domain. Hence, the PS-peptide-kinase domain interaction was used as a spatial constraint that in conjunction with torsional MD simulations was used to *de novo* identify potential C1a-kinase domain interfaces. Subsequently, mutagenesis of FRET sensors that probe the C1a-kinase domain interaction strength was used to experimentally validate the interaction site. Taken together, our study illustrates an integrated approach involving MD simulations and FRET sensors to map binding interfaces in multidomain kinases.

In the absence of activating stimuli, PKC resides in the cytosol. Ca^2+^ and DAG stimulation facilitate interactions between the regulatory domains and lipid membranes (C2-Ca^2+^ -- PS; C1-DAG). We have recently shown that the extent and rate of translocation can be tuned by varying the strength of the autoinhibitory interaction between the regulatory and kinase domains (14). Our study shows that SMKIs that influence regulatory interactions can allosterically impact the rate and levels of membrane recruitment of activated PKCα in live cells. Pretreatment of cells with BimI-like and staurosporine-like inhibitors enhances both the rate of membrane translocation in cells (Fig. 5*D*) and maximal membrane recruitment in cells and synthetic lipid bilayers (Fig. 5, *E* and *G*). In contrast, pretreatment with the ATP analog inhibitors sangivamycin and toyocamycin had no effect on either the rate of membrane translocation or maximal membrane recruitment compared with a DMSO control (Fig. 5, *E* and *G*). Given that membrane translocation of PKCs is a hallmark of their activation, the paradoxical enhancement of membrane recruitment by select SMKIs is an important feature that is currently overlooked in the selection of PKC-specific inhibitors. PKCs phosphorylate a range of kinase substrates, many of which are localized to cell membranes (EGFR (25), MARCKS (26), myelin basic protein (27), etc.). Hence, the aberrant localization of PKCα to the plasma membrane could increase the effective concentration of kinase-substrate interactions to confound the efficacy of kinase inhibitors. Further, SMKIs that influence PKC localization may impact pseudokinase functions that are mediated by numerous proteins that interact and are potentially scaffolded by this nodal kinase (11). While the specific effects of individual SMKIs are likely to be cell- and stimulus-dependent, our data provide a framework along with a systematic approach to evaluate the cellular mechanisms of PKC inhibition.

While our data are suggestive of a correlation between the C1a-kinase domain interaction and the rate and/or extent of membrane translocation, they support the role of other factors that influence SMKI-mediated membrane localization. PKCα translocation to the membrane is accompanied by intermolecular interactions, resulting in the formation of oligomers (16). Both BimI-like and staurosporine-like inhibitors were observed to enhance oligomer formation, as detected by bi-molecular FRET sensors whereas ATP analog inhibitors were not (Fig. S4*B*). However, we have previously shown that the size and distribution of PKCα oligomers is heterogeneous, complicating quantitative characterization of the relative influence of SMKIs (16). In previous work, we have shown that pretreatment of cells with BimI causes sustained membrane localization of PKCα in cells treated with LPA stimulation. Hence, we speculate that enhanced inter-PKC interactions by BimI and staurosporine-like inhibitors stabilizes PKC-membrane interactions resulting in enhanced maximal recruitment. Given that the release of regulatory domain autoinhibition enhances membrane accessibility of PKCα, SMKI-mediated effects on regulatory interactions and oligomerization are inherently intertwined and may cooperate to affect membrane localization. Finally, while PKCα membrane localization is likely influenced by other cellular factors such as its interaction with membrane-bound proteins, our findings with synthetic lipid bilayers reinforce the PKCα-intrinsic effects of SMKIs on membrane translocation.

The amino acid residues on the PKCα kinase domain that form the binding interface with the C1a regulatory domain are conserved across the PKC superfamily (Fig. 3*B*). However, outside of the GxGxFG motif in the G-loop, there is no sequential conservation across the broader AGC family, of which PKCs are a prominent member. This suggests that the conserved binding interface between the C1 and catalytic domains that we report here is unique to PKCs. Interestingly, closer examination of the interface on the kinase domain revealed a hydrophobic binding pocket containing the G-loop that is capable of accommodating a drug-like small molecule. Given that the G-loop forms the floor of this binding pocket, we termed this the G-loop pocket. Given that the G-loop is essential for positioning Mg^2+^-ATP for phosphoryl-transfer, small-molecule ligands that occupy the G-loop pocket are likely to influence G-loop conformation and consequently kinase activity. Analysis of the entire kinase domain in PKCα using fragment-based screening showed the G-loop pocket to be among the five most accessible pockets on the kinase domain for small-molecule ligands, including the nucleotide binding site. The lack of sequence conservation supports a potential role for the G-loop pocket as a kinase-selective target site for small-molecule allosteric modulators that merits further investigation in future studies.

## Experimental procedures

### Experimental methods

#### Reagents and constructs

PKCα constructs for *in vitro* assays were cloned into pBiex1 (Novagen) plasmid vector for expression in Sf9 insect cells (ThermoFisher). Constructs expressed in mammalian cells were cloned into pcDNA/FRT (ThermoFisher) plasmid vector as previously described (14). Constructs expressed in Sf9 contain a C-terminal FLAG tag and either a 10 nm ER/K helix with a FRET pair (mCerulean/mCitrine), an N-terminal mCerulean, or a C-terminal mCitrine. Constructs expressed in mammalian cells contain both an N-terminal mCer and a C-terminal mCit. In constructs containing the ER/K linker with separated domains, the linker was inserted between the regulatory domains (residues 1–293) and the catalytic domain (residues 335–672), the pseudo substrate (residues 1–31) and the catalytic domain, the C1a domain (residues 32–100) and the catalytic domain or the pseudo substrate, and c1a domains (residues 1–100) and the catalytic domain. Point mutations were inserted using site-directed mutagenesis (Pfu-Turbo, Agilent).

#### Protein expression and purification

PKCα constructs in pBiex1 vectors were transiently transfected in Sf9 insect cells as described earlier (28). Cultures were grown in Sf900-II media (ThermoFisher), and constructs were expressed using Escort IV transfection reagent (MilliporeSigma) and Opti-MEM I (ThermoFisher) for 3 days posttransfection. Cells were lysed in 20 mM Hepes (pH 7.5), 200 mM NaCl, 4 mM MgCl_2_, 0.5% sucrose (% w/v), 0.5% IGEPAL (% v/v), 2 mM DTT, 100 μg/ml of PMSF, 10 μg/ml of aprotinin, and 10 μg/ml of leupeptin. Lysate was incubated with anti-FLAG M2 affinity resin (MilliporeSigma) for at least 2 h. Resin was washed four times with 20 mM Hepes (pH 7.5), 150 mM NaCl, 10 mM MgCl2, 2 mM DTT, 100 μg/ml of PMSF, 10 μg/ml of aprotinin, and 10 μg/ml of leupeptin. Protein was eluted with FLAG peptide (MilliporeSigma). Protein concentrations were determined using mCitrine absorbance on a NanoDrop One (ThermoFisher) or mCitrine (excitation 490, emission 525 nm) or mCerulean (excitation 430, emission 475 nm) fluorescence on a on a FluoroMax-4 fluorometer (Horiba Scientific). For all experiments using purified PKCα, protein was centrifuged at >200,000*g* for 10 min to remove aggregates.

#### FRET measurements

All FRET measurements were performed in PKC buffer (20 mM Hepes (pH 7.5), 5 mM MgCl_2_, 0.5 mM EGTA, and 2 mM DTT). Intramolecular FRET assays used 25 nM protein, and intermolecular FRET assays used 40 nM mCerulean-tagged PKC plus 160 nM mCitrine-tagged PKC. Intramolecular measurements were taken both under basal and stimulating (1.5 mM CaCl_2_ and 3.2 μM PMA) conditions and were incubated at room temperature for 30 min before measurement. FRET spectra were measured by exciting mCerulean at 430 nm with an 8 nm bandpass and monitoring emission from 450 to 650 nm in 1 nm increments with a bandwidth of 4 nm. FRET ratio was calculated as the emission intensity at 525 nm divided by the emission intensity at 475 nm. Proteins were treated with inhibitors at the following concentrations: 1 μM BimI, 1 μM sotrastaurin, 1 μM staurosporine, 10 μM tetrahydrostaurosporine, 100 μM sangivamycin, or 100 μM toyocamycin. Samples were prepared in tubes precoated with 0.1 mg/ml bovine serum albumin to prevent protein from sticking to the sides of the tubes.

#### Mammalian cell culture and live cell imaging

Mammalian COS-7 cells (ATCC) were cultured in DMEM (ThermoFisher, #11960) supplemented with 10% fetal bovine serum (FBS, % v/v), 1% GlutaMAX (% v/v, ThermoFisher), and 20 mM Hepes (pH 7.5) at 37 °C with controlled humidity in 5% CO_2_ (% v/v). For live cell imaging, cells were transfected with fluorescent PKCα using X-tremeGENE HP (Roche Applied Science) and plated on 35 mm glass-bottom dishes (MatTek Corp). Immediately before imaging, cells were washed twice with Hepes imaging buffer (20 mM Hepes [pH 7.5], 2.5 mM MgCl_2_, 145 mM NaCl, 5 mM KCl) and exchanged into 0.5 ml of imaging buffer with the selected inhibitor or DMSO. Inhibitors were used at the following concentrations: 10 μM BimI, 10 μM sotrastaurin, 10 μM staurosporine, 10 μM tetrahydrostaurosporine, 100 μM sangivamycin, or 100 μM toyocamycin. Imaging was performed on a Nikon Eclipse Ti epifluorescence microscope with a 40x or 60x oil objective and a perfect focus system. Samples were illuminated through a dichroic containing a 500/40 nm excitation filter, a 520 long-pass dichroic, and a 535/30 nm emission filter to select for mCitrine fluorescence. Video was captured on a Photometrics Evolve camera at 0.5 frames per second with 400 ms exposure time for 15 min. After 20 frames (40 s), cells were stimulated with 0.5 ml of imaging buffer containing inhibitor and 0.2 mg/ml 1,2-dioctanoyl-*sn*-glycerol (DiC_8_; Avanti), for a final concentration of 0.1 mg/ml DiC_8_.

Images were analyzed with ImageJ (NIH) (29) as indicated Figure 4*A*. Briefly, this was done by measuring the average intensity of a cytoplasmic region around the nucleus (C) and of the membrane at the cell periphery (M) over time (t) for each cell. Normalized membrane intensity was calculated as (M_t_/C_t_) (C_t = 0_/M_t = 0_) to correct for photobleaching and cell to cell differences in total intensity. For each condition, ≥2 separate experiments were performed with ≥2 dishes and ≥2 cells per experiment. The three highest and three lowest normalized membrane intensity trajectories (or two highest and two lowest if N < 10 cells) for each condition were discarded as outliers. Following outlier removal there were N ≥ 6 cells for all conditions. I_max_ and I_slope_ were calculated for individual traces. For I_max_ calculations, traces were median filtered over five frames to eliminate any intensity spikes. I_slope_ was calculated using a linear fit to the first 100 s following stimulation. Data were fit using Origin (OriginLab Corporation).

#### *In vitro* membrane recruitment

Supported lipid bilayers containing mol% 5% diacylglycerol, 69.9% 1,2-dioleoyl-sn-glycero-3-phosphoethanolamine, 25% 1,2-dioleoyl-sn-glycero-3-phospho-L-serine, and 0.1% fluorescent PE were formed in a flow cell constructed with a channel of double-sided tape (3M) in between a cleaned glass coverslip and a glass slide. Coverslips were cleaned by soaking in acetone overnight. Excess acetone was removed by evaporation with dry nitrogen gas. Coverslips were then soaked in 7× Cleaning Solution (MP Biosciences), diluted 1:3 in deionized water for 1 h, and bath sonicated for 20 min. Coverslips were then washed in a stream of DI water for 5 min to remove excess detergent. The coverslips were then soaked in 100% ethanol for 1 h, followed by bath sonication for 20 min. Excess ethanol was removed by evaporation with dry nitrogen gas. Cleaned coverslips prepared using this protocol were used on the same day. Lipids in chloroform were combined, and the chloroform was evaporated using dry nitrogen gas. Residual chloroform was removed by incubating under vacuum for ≥30 min. To solubilize, lipids were resuspended to 4 mM in PKC buffer without DTT and incubated at 37 °C for ≥2 h. Small unilamellar vesicles were formed by tip sonication on ice (10–15 s bursts for 5–10 min) followed by bath sonication for 15 min. Lipids were used the same day. Bilayers were formed by flowing lipids into the flow cell and incubating for ≥30 min. Membrane fluidity was verified for every flow cell using fluorescence recovery after photobleaching. PKCα binding was measured by flowing in 100 nM mCerulean-tagged PKC in PKC buffer with inhibitor, 2 mM CaCl_2_, or 1 mM ATP and measuring mCerulean intensity at the membrane surface. Inhibitors were used at the following concentrations: 10 μM sotrastaurin, 10 μM tetrahydrostaurosporine, or 100 μM sangivamycin. Imaging was performed on a Nikon Eclipse Ti epifluorescence microscope with a 100x oil objective and a perfect focus system. Samples were illuminated through a dichroic containing a 436/40 nm excitation filter, a 455 long-pass dichroic, and a 480/30 nm emission filter to select for mCerulean (PKCα) fluorescence or containing a 545/30 nm excitation filter, a 570 long-pass dichroic, and a 610/75 emission filter to select for rhodamine (bilayer) fluorescence. Movies were captured on a Photometrics Evolve camera at 10 frames per second for 15 min with gain and multiplier held constant for all PKCα movies on a given day. Bilayer fluorescence recovery after photobleaching was measured both before and after addition of PKCα. PKCα intensity was measured in eight regions for each flow cell. Background intensities were measured by imaging a bilayer with the mCerulean filters before adding PKCα. Intensities for each flow cell were averaged and background subtracted and normalized to the ATP, no inhibitor condition for each day to correct for day-to-day lipid and intensity variability. Data were analyzed using ImageJ (NIH) (29). For each condition ≥3 separate experiments were performed using different protein and lipid preparations.

### Computational methods

#### Atomistic model of the C1a-kinase domain binding interface

Fig. S1 sequentially outlines the hierarchical, multiscale MD simulation procedure we used to identify (Steps 1–6) the C1a-kinase domain interface.

##### Step 1: torsional angle molecular dynamics simulations with replica exchange for coarse grain identification of putative C1a-kinase domain interactions

Our goal was to derive a structural model for the C1a interaction with the kinase domain (Fig. S1*A*). However, there is currently no crystal structure of the full-length PKCα. In our previous work, we started from the crystal structure of the kinase domain of PKCα (PDB ID: 3IW4) and derived a model of the kinase domain interacting with PS using GNEIMO running replica exchange MD (REMD) simulations (18, 30). We also used the crystal structure of the kinase domain of PKCα that includes the C1b domain from the crystal structure of the full-length PKCβII (PDB ID: 3PFQ) (31). Here, we used these two previous structures as a starting point to predict the binding site for C1a on the kinase domain. Using the C1b and PS docked onto the kinase domain as constraints, we built in the two linkers V1 (connecting PS to C1a) and V1’ (connecting C1a to C1b) in extended chain conformations pointed away from kinase domain. The C1a was therefore suspended in the middle of these two linkers, about 35 Å away from kinase domain mass center, in two different orientations, and both of which had no direct contact with kinase domain. We then performed torsion angle molecular dynamics simulations GNEIMO combined with temperature based replica exchange (32).

##### Step 2: GNEIMO-REMD annealing protocol

To fold the structure of the linkers and optimize the C1 binding to the kinase domain, we performed 400,000 annealing cycles using GNEIMO-REMD-Rosetta torsion MD simulations using the protocol described below (Fig. S1*B*). GNEIMO is the internal coordinate MD package that has been combined with Rosetta software (33) for refining protein structures (34). The Rosetta module was used for force field and side-chain repacking after each annealing cycle. Each annealing cycle involved REMD using GNEIMO torsional MD with a temperature range of 275 to 450K. Each annealing cycle consisted of a side-chain rotamer repacking of all the residues in the whole complex using the Rosetta *PackRotamersMover* module, an all-atom minimization using the *CartesianMinimizer*, followed by the GNEIMO-REMD-Rosetta torsion MD simulation run with four replicas. The GNEIMO-REMD torsion angle simulations were done using the multibody dynamic model, where the backbone torsional angles of all the secondary structure elements were treated as rigid and all other torsion angles as flexible. We used an integration step size of 1 fs. We used the Talaris 2014 with Lazaridis-Karplus implicit solvent model and a distance-dependent dielectric function, a variant of the Lobatto integrator within the GNEIMO module3, and 0.5 ps of Nose-Hoover constant (τ) at constant temperature (35). We performed 400,000 annealing cycles with snapshot of the lowest energy conformation from each cycle stored. This amounts to 12 μs of total simulation time.

##### Step 3: identifying potential interaction interfaces

GNEIMO-REMD conformations that showed C1a interacting directly with the kinase domain were selected for further analysis (Fig. S1*C*). These conformations were clustered by RMSD in coordinates of the Cα atoms of the whole complex with a cutoff of 0.9 Å and identified 137 conformation clusters. We saved the representative frame of each conformational cluster and deleted the V1’ and C1b domain for further refinement of the models. All selected kinase domain-C1a bound models were energy minimized. We then calculated the energy of the C1a-kinase domain complex for all these representative conformations. The top five best scoring structures sorted by energy of the CD-C1a complex were chosen for analysis of interface interaction energy as described below.

##### Step 4: selecting C1a-kinase domain interaction structural models based on interface interaction energy

All selected kinase domain-C1a–bound models were energy minimized and the binding interface interaction energies were evaluated by Rosetta (36) (Fig. S1*D*). During the interface interaction energy calculation, every residue in the C1a domain (residues 38–96) was considered interface 1, and every residue in the kinase domain (residues 333–666) was considered as interface 2. A short energy minimization was performed using the minimize.static.linuxgccrelease protocol, with minimization method set to lbfgs_armijo_nonmonotone, and tolerance set to 0.001. The interface energy was calculated using the interface_energy.static.linuxgccrelease protocol. This analysis identifies models 1 and 5 as candidate strong interactions.

##### Step 5: potential to disrupt the C1a-kinase domain interaction using mutagenesis

As a complementary approach to rank order our models, we evaluated changes in interaction energy upon mutating each interface residue to all remaining 19 amino acid types in all five of the kinase domain structural models (Fig. S1*E*). Models with more favorable interface interaction energy are likely to show greater changes upon mutagenesis, reinforcing their selection for experimental validation. We calculated the interaction energies of the C1a domain (residue number range 38–96) with the catalytic domain (residue range 333–666) using Rosetta mutation protocol (37). We first performed energy minimization using minimize.static.linuxgccrelease protocol, with minimization method set to lbfgs_armijo_nonmonotone and tolerance set to 0.001. The interface energy was calculated by interface_energy.static.linuxgccrelease protocol. We mutated every residue on C1a located in the interface of kinase domain-C1a to all other 20 residues in the top five models by mutate.static.linuxgccrelease protocol in Rosetta and then calculated the interface interaction energy difference between the mutant and the WT. The interface interaction energy was calculated using the same protocol with Rosetta (36). Histograms of changes in interface energy upon mutation of each interface residue on C1a to 20 amino acid types were used to identify which residue positions were sensitive to mutations in models 1 to 5.

##### Step 6: identifying triple mutants for experimental testing of computational models

From all of the single mutations, we chose the top three single mutations that caused the greatest change in the interface interaction energy upon mutation (values listed in Fig. S1*F*). To minimize experimental iteration and enhance mutant effects, triple mutant combinations were chosen for experimental testing based on the difference of interface interaction energy on kinase domain-C1a interaction.

#### Refinement of C1a-kinase domain binding interface

The energies calculated from Rosetta and GNEIMO-REMD conformations are approximate because there is no explicit desolvation in these calculations. Therefore to correlate the interface energies with changes in FRET sensor intensity measurements, we refined the structural models of conformation of C1a-kinase domain by performing all-atom MD simulations in explicit solvent. To this end, we performed 1 μs of all-atom MD simulations (see protocol) in explicit solvent on the model 5 starting from the GNEIMO-REMD Rosetta energy minimized structure, for the WT and the four mutants proposed in previous step, using GROMACS MD package (38). For the WT and each mutant system, we calculated the average C1a-kinase domain interface interaction energy (averaged over the last 500 ns of the simulation trajectory) from each trajectory. The interaction energy between C1a and kinase domain was calculated by mmpbsa method that takes the desolvation penalty into account (39).

#### All-atom MD simulation protocol

We performed all-atom MD simulations for all the binding models, mutant, protein–inhibitor complex structures in explicit solvent with the GROMACS2016 package (38) with GROMOS 54a7 force field (40). Each of the prepared structures were solvated in a 90 Å^3^ cubic TIP3 water box, then the system was neutralized with 0.15 M NaCl. The LINCS algorithm was applied on all bonds and angles of water molecules with a 2 fs time step used for integration. We used a cut-off of 12 Å for nonbond interactions and a particle mesh Ewald method for long range van der Waals interaction. Each system was slowly heated from 0 K to 310 K in NVT ensemble during a 1 ns heating process. The temperature was maintained using a Nosé-Hoover thermostat (41). We equilibrated the system with harmonic position restraints on all protein and ligand heavy atoms for 30 ns. The constrained force constant was set to 5 kcal/mol-Å^2^ initially and gradually reduced to 0 kcal/mol-Å^2^ with a 1 kcal/mol-Å^2^ per 5 ns window. The pressure was controlled by the Parrinello-Rahman method (42), and the simulation system was coupled to a 1 bar bath. The last frame from the equilibrium process was used as the initial conformation for production simulations. We initiated five different production runs, each 200 ns long, using five random seeds for velocity initialization. The last 100 ns of each production run was aggregated and used for analysis.

#### All-atom MD simulations of C1a-kinase domain-PS complex with inhibitors

To better understand the effect of ATP-competitive SMKIs on the PS–C1a-kinase domain complex, we modeled six ATP competitive inhibitors in the C1a-kinase domain complex model. The most representative structure from WT simulation in previous section was taken as initial configuration for modeling inhibitor–protein complex. The six inhibitors, BimI, sotrastaurin, staurosporine, tetrahydrostaurosporine, sangivamycin, and toyocamycin, were docked into the ATP binding pocket in the C1a-kinase domain-PS model chosen from the previous section. We performed Glide flexible ligand docking (Schrödinger Release 2018-2 (43, 44, 45)) using a clash score of 100 kcal/mol. We then chose the best docked pose for the six inhibitors that showed similar poses to those in the crystal structures of the kinase domain bound to each inhibitor in other kinase-inhibitor complex structures. The generated protein–ligand complex was energy minimized using the GROMACS package. The force field parameters for inhibitors were taken from previous models (42). During the 1000 steps minimization process with steepest descent method, a restraint force of 1000 kJ/mol was applied on all protein backbone heavy atoms and ligand heavy atoms, and only the protein sidechain and all hydrogens were treated as flexible. These minimized structures were then taken as the initial structures for further 1 μs all atom MD simulations using the protocol described above.

The kinase domain-C1a interaction energies in the presence of different inhibitors were calculated using the last 500 ns of each production run. The kinase domain-C1a interaction energy was calculated by mmpbsa method (39). The structures shown in Figure 4*A* are representative structures of the most occupied conformation cluster extracted for each inhibitor from these simulations. The conformation clustering was done using the 1 μs long aggregated trajectories for each inhibitor. A RMSD cutoff of 1.5 Å for the Cα atoms was used in GROMACS to do this clustering. The representative structures were aligned by the Cα atoms of the whole C1a–CD complex and shown in Figure 4*A*.

#### Simulating the displacement of C1a from the kinase domain when bound to ATP competitive inhibitors

Some of the SMKIs showed displacement of the C1a from the kinase domain as the result of inhibitors binding. To simulate the early events of this dissociation or weakening of kinase domain-C1a interactions, we performed torsion angle GNEIMO-REMD simulations on the apo and BimI bound C1a-kinase domain-PS models derived in the previous section. We performed 200,000 cycles of GNEIMO-REMD annealing simulations totaling to 6 μs each for BimI and apo conditions. The trajectories of the GNEIMO simulations showed a 5 Å displacement of the center of mass of C1a from the G-loop when BimI was bound in the ATP binding site compared with the apo protein with nothing bound. The resulting GNEIMO trajectory was subjected to conformational clustering analysis. The representative structure from the most populated conformational cluster of the BimI bound C1-kinase domain-PS system was compared with that of the apo system.

#### Sequence alignment

We performed amino acid sequence alignment of the N-lobe of the kinase domain of PKCα to check if the residues on the kinase domain lining the C1a binding pocket are conserved across AGC kinases. The sequence alignment was performed using Clustal Omega (46).

#### Identification of putative small molecule binding site in the C1a–CD interface

We used the computational method developed by Li *et al.* (21) called FBS to identify putative small molecule binding sites in proteins. We docked a 60,000 molecule library of diverse small molecules to the entire protein structure using the Glide docking program. We then clustered the regions with the highest docked ligand atom density obtained from docking. Previously, we have shown that these regions correspond to protein–protein interacting interfaces (21). The experimentally determined hotspot residues for each protein–protein complex, cluster near the best scoring druggable binding sites identified by FBS. We used FBS here and identified a putative small molecule binding site near the surface of C1a-kinase domain interactions.

## Data availability

The starting structures and all-atom MD trajectories are available upon request (nvaidehi@coh.org). All remaining data are contained within the manuscript.

## Conflict of interests

The authors declare no conflicts of interest in regard to this manuscript.
