# Supplementary material for: Kinase inhibitors allosterically disrupt a regulatory interaction to enhance PKCα membrane translocation
Source: J Biol Chem. 2021 Jan 26;296:100339. doi: 10.1016/j.jbc.2021.100339 (PMC7949123; doi:10.1016/j.jbc.2021.100339)
Supplement: Supplemental Figures S1–S4 [file mmc1.pdf]

**Supporting Information**

**for:**

# Kinase inhibitors allosterically disrupt a regulatory interaction to enhance PKC$\alpha$ membrane translocation

Lisa G. Lippert, Ning Ma, Michael Ritt, Abhinandan Jain, Nagarajan Vaidehi, Sivaraj Sivaramakrishnan

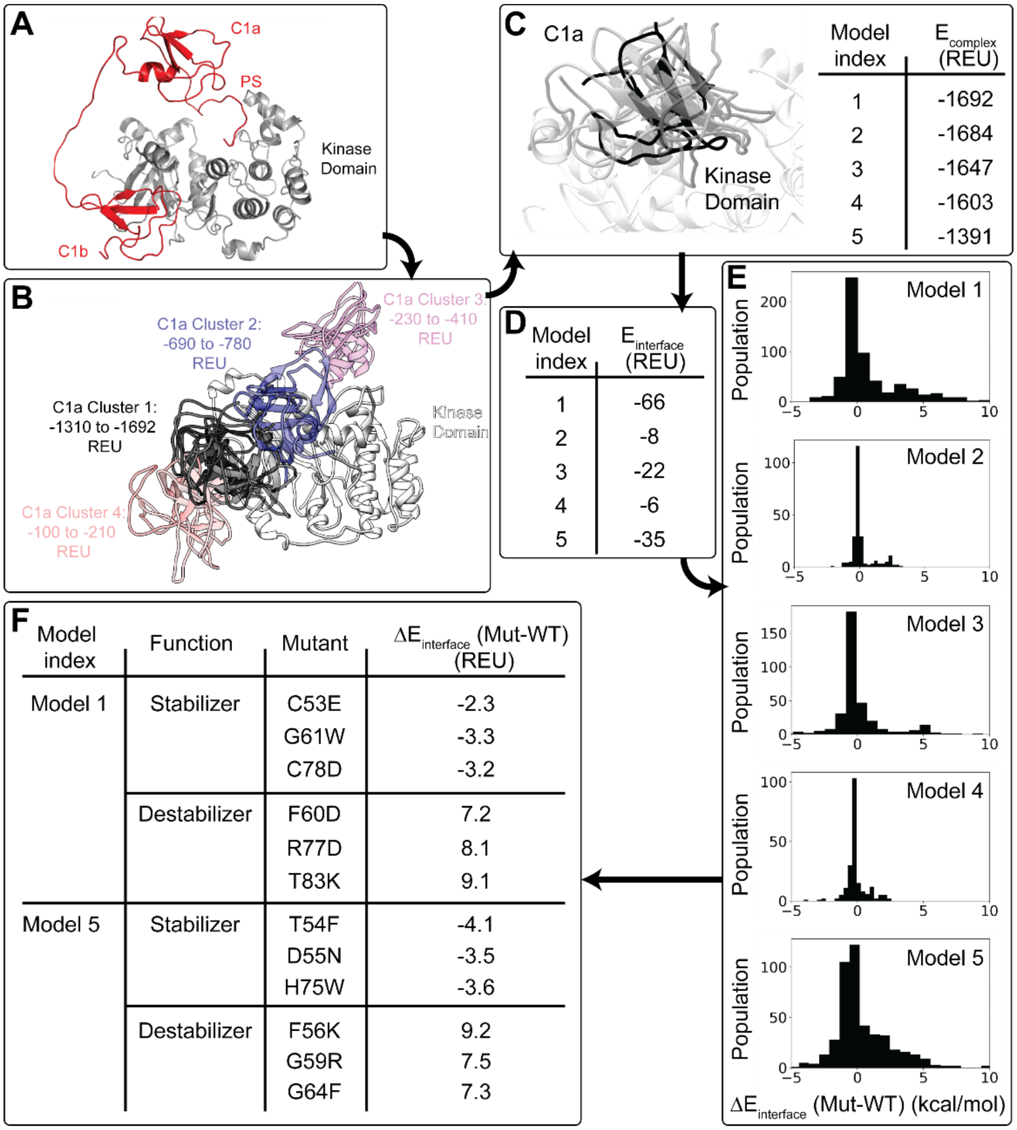


**S. Figure 1 –Multi-scale MD simulation procedure for generating structural models of C1a -CD interactions (A)** Construct initial models of C1a interacting with kinase domain (CD) in PKCα with constraints imposed from the model of PKCα with pseudosubstrate bound built in our previous work (Lee et al JBC 2014). **(B)** We generated 137 conformation clusters (clustered by RMSD in backbone coordinates of the PKC) using aggregated GNEIMO-torsional REMD simulation trajectories from 400,000 annealing cycles (see Methods). The representative structures extracted from the top 4 most populated conformation clusters are shown here. The total energy of the C1a-CD complexes calculated using Rosetta forcefield are shown in the figure in Rosetta energy units (REU). **(C)** We chose the top five C1a-CD models from conformation cluster 1 based on the complex energy shown in the table here. **(D)** Models 1 and 5 from top 5 models showed favorable interface interaction energies between C1a and the kinase domain calculated using Rosetta forcefield. **(E)** We modeled mutations at every interface residue to all 19 other amino acid in all five models using Rosetta Mutation module. Models 1 and 5 showed the maximum number of residues positions that were sensitive to mutations showing significant changes in the interface energy of the mutant upon mutation. **(F)** We identified the top 3 mutations that showed the biggest positive/negative change in C1a-CD interface interaction energy calculated using Rosetta ($\Delta E_{interface}$). We merged the top 3 mutants that caused the most significant change in interaction energy and proceeded to test them for activity experimentally.

A

| Cluster Index | Population | Mean Score | Min Score |
|---|---|---|---|
| 1 | 3744 | -4.37886 | -7.63434 |
| 2 | 2211 | -3.93254 | -7.04821 |
| 3 | 348 | -4.76127 | -7.12800 |
| 4 | 339 | -3.83635 | -5.97992 |
| 5 | 363 | -3.86682 | -6.22897 |

B

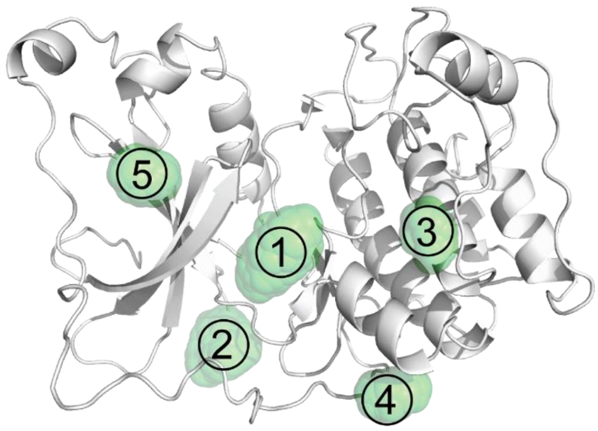


C

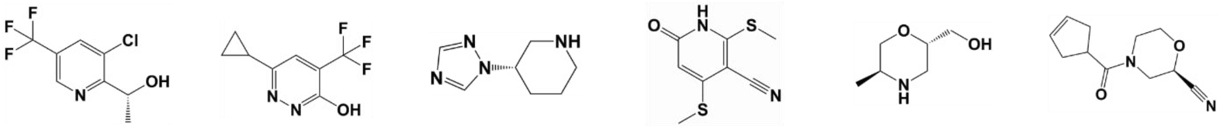

01390372 20285798 39090917 40487564 76994719 78210742

**S. Figure 2 – Identification of potential small molecule binding pockets on the surface of the kinase domain (A)** The number of small molecules (out of 60,000 small molecules docked) binding to each of the indicated binding pockets (population), the average docking score of all fragments in each pocket (mean score) and the best binding score (min score) from each pocket from the top 5 druggable small molecule binding pockets identified by *FindBindSite* (FBS). **(B)** Green spheres representing the position of the top 5 druggable pockets on the surface of the kinase domain of PKCα identified by FBS. **(C)** Six representative small molecule fragments which can bind in the C1a-CD interaction site pocket, identified by FBS.

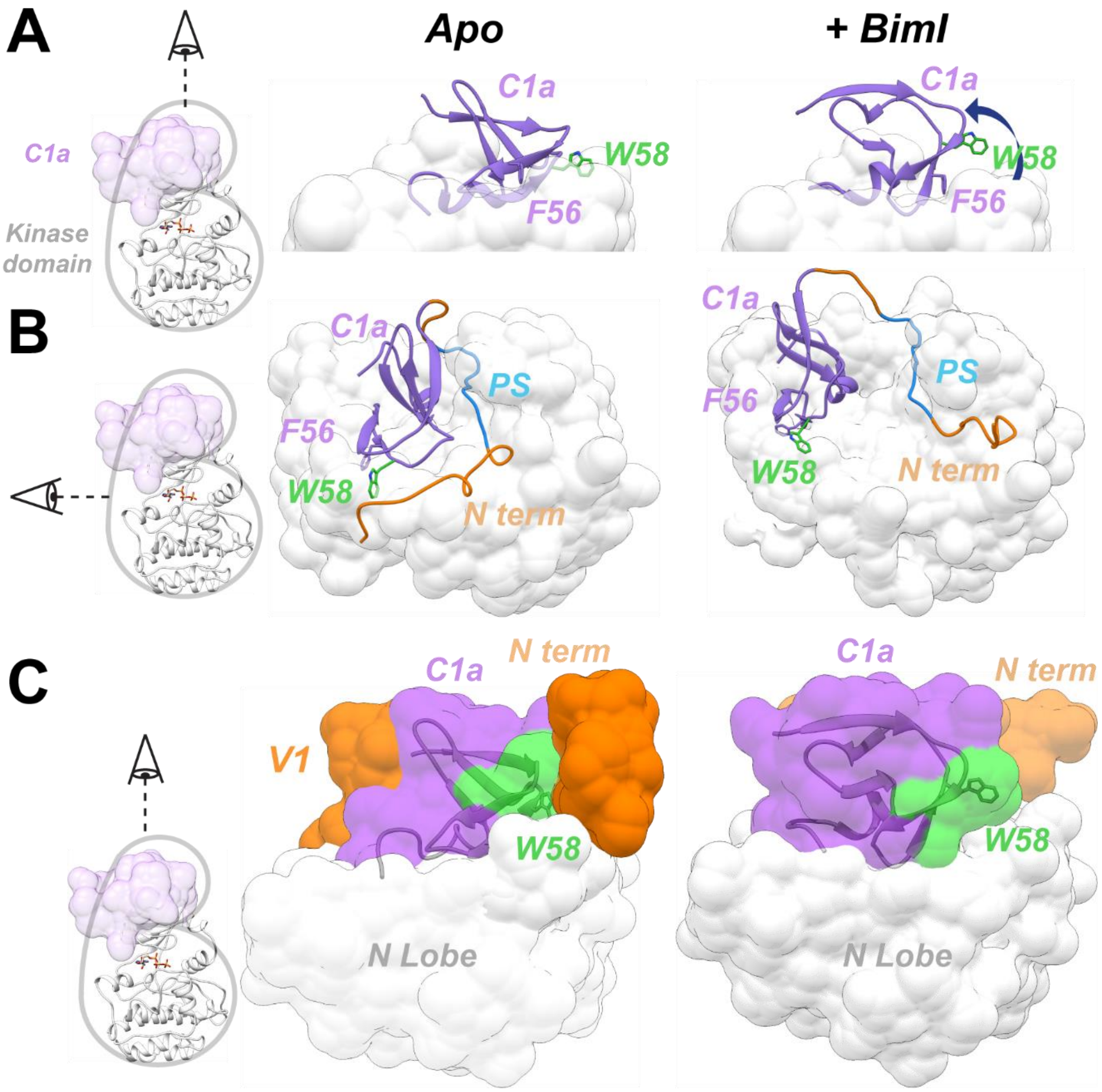


**S. Figure 3 - Mechanistic insight into the weakening of C1a-kinase domain interactions by Bim1 from the MD simulations. (A)** Surface rendering of kinase domain (white) interacting with ribbon representation of C1a (purple) depicts solvent exposure of putative DAG binding residues (F56 and W58) upon BimI binding to the kinase domain. **(B, C)** Coordinated changes in C1a (purple), pseudosubstrate (PS; cyan), V1 linker region (orange), and N-terminus of kinase domain (dark orange) relative to the kinase domain (white surface) lead to exposure of the DAG binding site. **(A, C)** Top view; **(B)** side view.

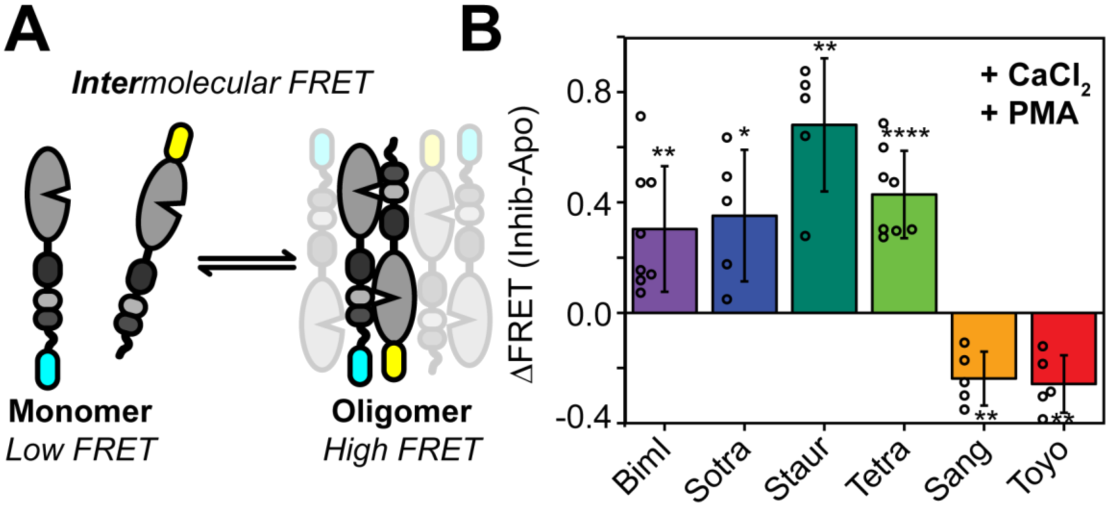


**S. Figure 4 – Inhibitors enhance oligomerization (A)** Cartoon depiction of PKC constructs used to detect intermolecular interactions. PKC constructs were tagged either on their N-termini with an mCerulean or on their C-termini with an mCitrine. FRET can only be observed when two separate PKC molecules form a dimer or higher order oligomer. **(B)** Change in oligomerization in the presence of the indicated inhibitors. Oligomer formation was measured using FRET in a bimolecular assay under stimulating conditions (calcium and PMA). FRET is plotted as the change in FRET ratio relative to the no inhibitor condition. Error bars show S.D. Circles indicate individual repeats. N $\geq$ 5 measurements from different protein preparations Significance was determined using a paired t-test of the raw FRET ratios relative to no inhibitor where **** indicates $p < 0.0001$, ** indicates $p < 0.01$, and * indicates $p < 0.05$.
